# Ocular Manifestations Following Encephalitis Lethargica
*Barrett Roué Prize Essay.


**Published:** 1928

**Authors:** A. Michael Critchley

**Affiliations:** Casualty Officer, Royal Free Hospital; late House Surgeon, Bristol General Hospital


### OCULAR MANIFESTATIONS FOLLOWING ENCEPHALITIS LETHARGICA.*

BY

A. Michael Critchley, M.B., Ch.B. Bristol,
*Casualty Officer, Royal Free Hospital ; late House Surgeon, Bristol General Hospital.*

---

Encephalitis Lethargica, from its initial phase to the final stages of chronic encephalitis, presents a large variety of ocular signs and symptoms. These eye changes are very varied, yet some are so constant as to be most valuable aids to diagnosis both in the early and late stages of the disease. The following article is a résumé of the ocular manifestations of encephalitis lethargica based upon personal observations of 72 patients.

### A. Pupillary Abnormalities.

(1) *Unequal Pupils.*—Anisocoria is a fairly common occurrence both in acute and chronic encephalitis. Duncan[1] found pupillary inequality in 29 of his series of 75 cases, associated in nearly every instance with some other defect, such as irregularity or impaired reactions.

(2) *Irregular Pupils.*—This group includes cases of non-circular and eccentrically placed pupils. Such irregularities have frequently been described, though present but rarely in my series.

* Barrett Roué Prize Essay.

(3) *Argyll-Robertson Pupils.*—The syndrome of a pupil reacting on accommodation, but not to light, is sometimes found in this disease, especially in the late stages, and unless other physical signs are present, difficulty in diagnosis may quite easily arise. The occurrence in encephalitis of Argyll-Robertson pupils, together with absent knee-jerks, was first recorded in 1919 by Naëf[2] of Munich. Since then it has been occasionally reported.

(4) *Sluggish Pupils.*—In certain cases of chronic encephalitis the pupillary reactions, though present, may be abnormally sluggish in their time reactions. Moreover, the amplitude of the reflex dilatation and contraction of the pupil may be abnormally small. Duncan found this impaired briskness of reaction in 28 per cent. of his cases.

(5) *Failure to React on Accommodation.*—One of the most characteristic features of a typical post-encephalitic is an inability of the pupil to react on accommodation, whilst the reaction to light remains brisk. This abnormality is found in all types of chronic encephalitis, and is of immense value from a medico-legal standpoint, as it may be one of the few signs present in a case of conduct alteration following an attack of encephalitis lethargica. In Duncan's series pupillary reaction on accommodation was lost in 49 per cent., a figure which is low compared with my own.

### B. DISORDERS OF THE EYELIDS.

Almost every case of chronic encephalitis presents some changes in the eyelids, and a diagnosis of the disease can frequently be made from observation of these phenomena alone.

1. *Ptosis.*—Ptosis has been frequently described in all phases of this malady, and may in any stage be either unilateral or bilateral. It is most commonly seen at the onset of the disease. The Government Report[3] in 1918 on epidemic encephalitis states that over 50 per cent. of cases had ptosis at the onset.

2. *Lid Retraction.*—The typical staring gaze of the post-encephalitic Parkinsonian is often accentuated by a definite retraction of the eyelids. It gives a look of exophthalmos to the patient, and von Graefe's and Stellwag's signs may be positive. This retraction is sometimes variable, and is not always present. It may actually alternate with ptosis, so that at one examination the patient may have ptosis, and when next seen lid retraction may be marked.

3. *Blepharoclonus.*—This sign is said to be present when, on closing the eyelids, the lids are thrown into a series of rapid clonic movements, of fairly large amplitude. These clonic movements are regular in their rate and amplitude, and persist so long as voluntary closure of the eyelids is maintained. When the eyelids are closed in sleep, no movements are seen. According to Hall[4] this tremor is brought out and exaggerated by an attempt on the part of the patient to open his eyes quickly. This flickering of the closed eyelids resembles that seen in neurasthenia, but differs in being much coarser.

4. *Diminished Blinking.*—Parkinsonians are found to blink less often than the normal individual. As a result the surface of the eyeball is not adequately lubricated, and foreign bodies are apt to injure the conjunctiva ; hence blepharitis and conjunctivitis may be found as complications of this state. The infrequent blinking is another factor in the production of the typical Parkinsonian stare. The impression thus given is one of fixity of gaze; suggesting, in turn, fixity of ideation or mental concentration. Such an impression may be quite false, for in the post-encephalitic the facial expression frequently belies the true emotional content.

5. *Blinking Sign on Lateral Deviation.*—This phenomenon, though not unknown in normal individuals, is highly characteristic of the Parkinsonian syndrome. Kinnier Wilson,[5] who first reported it, described it as follows in his Croonian Lecture:—"When the Parkinsonian is asked to make a series of successive right and left conjugate deviating movements of the eyeballs . . . he very frequently blinks his eyelids as the eyes pass from one extreme lateral position to the other. The blinking movement, of which he appears to be unaware, is sometimes quick, sometimes slow and deliberate. In more than one case a quick double eyelid flicker has been distinctly and repeatedly observed." This sign may depend on the dryness of the conjunctiva through diminished blinking, necessitating the act of blinking to lubricate the eyes before they can move easily.

6. *On Convergence the Eyelids remain up.*—Normally when a person looks at a near object, the eyes converge, the pupils contract, and the upper lids gracefully lower. In chronic encephalitis each component of this co-ordinate movement complex is upset: the eyes do not converge, accommodation of the pupil is often lost, and the eyelids remain elevated.

### C. DISORDERS OF MOVEMENTS.

1. *Limitation of Movement.* The majority of post-encephalitics are unable to converge, which is of course a limitation in the range of movements, but it is so frequent and important a sign that it is reserved for a separate paragraph. In addition to inability to converge, some cases are unable to move their eyes in a particular direction. This limitation of full ocular movement may only occur in lateral deviation, less rarely on upward and downward deviation.

2. *Punctuate Movements.*— Generalised rigidity, often of the "cog-wheel" type, together with a slowness of movement (or bradykinesia), is generally found in Parkinsonism. A similar type of movement is present in the voluntary muscles of the eyeball, so that the eye moves in a characteristically jerky fashion. This phenomenon is best observed during lateral movements of the eyes. It may possibly be the expression of a similar hypertonus in the extrinsic ocular muscles.

3. *Diplopia.*—Often the only clue to the date of onset of encephalitis lethargica is a history of "influenza" or a "cold," which was accompanied by a brief spell of double vision. This diplopia is caused by a disturbance of synergic action of the muscles rather than by isolated paralysis or paralyses. If the diplopia persists, and is present in the chronic stages, there is then found some paralysis, though rarely associated with strabismus. Chambers[6] has pointed out that in some of these cases the diplopia is only present with distant fixation. There is much to suggest that distant fixation is an active function carried out by the simultaneous and synergic contraction of both external recti, with a corresponding relaxation of the internal recti. Diplopia may in such cases, therefore, be the expression of a weakness of divergence.

4. *Strabismus.*—Strabismus is quite common in the acute stages, but in the chronic phase is less often seen. Frequently the squint only occurs when looking in a certain direction, and is found to be due to a mild degree of paresis of a single muscle.

5. *Paralysis of Convergence.*—One of the chief distinctions between Parkinsonism due to encephalitis lethargica and that due to paralysis agitans is that in the latter there is never any difficulty in converging, unless the subject be very advanced in years, whilst in the former convergence is lost. On attempting to converge, the eyes may look towards the nose temporarily, but almost immediately one eye will deviate outwards again.

6. *Oculo-gyral Crises* (*Tonic Eye Fits*).—These crises are of immense interest, and the theories as to their pathogenesis have caused considerable controversy. The crises consist in a spasmodic conjugate deviation of the eyes usually in an upward direction, coming on in paroxysms, and lasting for a period varying from a few seconds to hours. They are often accompanied by, or rather are part of, a generalised convulsion. These attacks are a late complication of epidemic encephalitis, and are not common. Bing[7] found only three cases of tonic eye fits amongst three hundred encephalitics; Wimmer[8] found five cases from a similar number of patients. There is some evidence to suggest that they are becoming commoner. A fuller discussion of these phenomena appears later.

### INCIDENCE OF OCULAR SYMPTOMS.

The following table shows the number of times each ocular manifestation was found in my series of 72 chronic encephalitics, and the percentage incidence as compared with Young's[9] tables :—

TABLE I.

| | Number of Cases. | Per-centage. | Young's percentage |
|---|---|---|---|
| Blepharoclonus .. .. .. .. .. | 69 | 95·8 | 92 |
| Punctate eye movements .. .. | 64 | 88·8 | 64 |
| Diminished blinking .. .. .. | 63 | 87·5 | — |
| Loss of convergence .. .. .. | 59 | 82·0 | 81 |
| Loss of accommodation .. .. | 50 | 69·5 | 64 |
| "Blinking sign" .. .. .. .. | 47 | 65·3 | 67 |
| Diplopia .. .. .. .. .. .. | 37 | 51·4 | 41 |
| Lid retraction .. .. .. .. | 19 | 26·4 | — |
| Ptosis .. .. .. .. .. .. | 15 | 20·8 | — |
| Unequal pupils .. .. .. .. | 9 | 12·5 | — |
| Limitation of movements .. .. | 9 | 12·5 | — |
| Pupil reaction poor or lost .. .. | 8 | 11·1 | 13 |
| Strabismus .. .. .. .. .. | 7 | 9·6 | — |
| Sluggish pupils .. .. .. .. | 6 | 8·3 | — |
| Oculogyral crises .. .. .. .. | 4 | 5·6 | — |
| Irregular pupils .. .. .. .. | 4 | 5·6 | — |
| Argyll-Robertson pupils .. .. | 2 | 2·8 | — |
| Nystagmus .. .. .. .. .. | — | 2·8 | — |
| Pupil reaction to light poor or lost | — | — | — |

The ocular signs may accompany any type of chronic encephalitis. My cases were of the following types :—

TABLE II.

| | |
|---|---|
| Parkinsonism .. .. .. .. | 53 |
| Parkinsonism with respiratory disorder | 10 |
| Parkinsonism with conduct change .. | 4 |
| Parkinsonism with conduct change and palilalia .. .. .. .. | 1 |
| Sleep inversion only .. .. .. | 1 |
| Conduct change only .. .. .. | 4 |

The value of the less obvious and finer ocular signs in the diagnosis of chronic encephalitis in non-Parkinsonians will be seen from the following cases :—

*Cases of Conduct Change only.*

1. Female, age 15 years. Accommodation and convergence normal. No diplopia. Blinking normal. Punctate eye movements. Definite blepharoclonus.

2. Female, age 10 years. Accommodation poor, convergence lost. Eye movements even. No diplopia. Blinking normal. Blepharoclonus present.

3. Male, age 15 years. Accommodation and convergence lost. Eye movements jerky. No diplopia. Blinking less frequent. Blepharoclonus present.

4. Male, age 16 years. No accommodation, no convergence. Ocular movements smooth, but limitation of movements on looking towards left. No diplopia. Blinking less frequent. Definite tremor of eyelids.

*Case of Sleep Inversion only.*

Female, age 21 years. Accommodation and convergence lost. No jerky movements. Right external strabismus. Diplopia. Blinking normal. Blepharoclonus.

### OCULOGYRAL CRISES.

Until the last three or four years these crises were practically unknown, possibly because they are rather late sequelæ of encephalitis. To the best of my knowledge the first case was recorded in November, 1923, by Hohman.[10] His patient was a street-car conductor, who was discharged from his employment because "his eyes turned up, and he could not get them down." Since then many cases of this interesting phenomenon have been recorded.

As previously mentioned, the ocular crises consist in spasms of conjugate deviation of the eyes, usually in an upward direction. The fits may occur at any time, but most commonly when the patient is tired, as in one of my cases, or under the influence of emotional stress. As a rule the attacks are sudden in onset, but sometimes the patient has premonitory symptoms. During the fit itself nystagmoid jerkings of the eyes may be observed, and occasionally, as in my third case, a definite spasm of the orbiculares palpebrarum may occur, so that the patient is unable to open his eyes.

The oculogyral crisis is found only in the Parkinsonian syndrome, and may form part of a more generalised tonic fit. Thus simultaneous spasms of the arm, facial and respiratory muscles, as described by Bing, may be associated with the ocular spasms (Case 1). During the fit the patient does not lose consciousness, but may complain of visual hallucinations. Pascheff[11] describes a "tic" or syndrome characterised by periodic upward deviation of the eyes accompanied by visual pseudo-hallucinations—"pseudo," because the patient knows the hallucinations are unreal. The patients may see "red," possibly due to venous stasis, or may experience pain. Wimmer records one case in which the patient always complained of "imperative hallucinations, urging the patient to commit suicide." The paroxysms may last from a few seconds to the greater part of a day. After a protracted crisis the patient may fall into a sleep of exhaustion; much more rarely a short paroxysm may be followed by an attack of irresistible sleep from which he is promptly aroused. Such sleep fulfils the postulates of Adie, and is probably narcoleptic in nature. Lastly, the patient may find by experience that sleep is the only measure which will terminate the oculogyral crisis, which otherwise persists until bedtime. Such was the case in my second patient.

**Case 1.**—Female, aged 21. Acute encephalitis 1920. Has developed into a typical Parkinsonian, with respiratory disorder, frequently yawning, and occasionally giving a loud, prolonged sigh. Her pupils are equal, regular and central, reacting to light but not on accommodation; cannot converge. Her lids are retracted, and on closing them there is blepharo-clonus. Blinking is less frequent, and she blinks on looking to the side. When looking to the right or to the left her eyes move before her head. For the last twelve months she has been subject to frequent oculogyric crises, occurring once every third day during her stay in hospital. When she is tired or excited she gets premonitory feelings that she must look up; finally, she is impelled to gaze in an upward direction, and is then unable to lower her eyes. An attack lasts from half a minute to half an hour. During these crises she cannot close her eyes, which lacrimate freely. Coarse nystagmus in a lateral direction occurs. The eyelids are retracted, her forehead wrinkled, her head hyperextended, and there is a tremor of the cheeks. The mouth remains open, and she gives a yawn and a sigh every few minutes. Her whole body is rigid and tremulous, especially marked on the left side. The patient is very agitated by these attacks, which at the time cause her pain at the back of the eyes, in the frontal region, and sometimes in the ear. All objects appear to be moving from side to side, but there are no visual hallucinations.

When an attack comes on she lies down in a darkened room with something thrown over her eyes, and after about fifteen minutes she becomes able to lower her eyes. She then sleeps for several hours, as she feels too exhausted to pursue her normal occupation.

**Case 2.**—Male, aged 25. This patient is a soldier, and gives no history of an acute attack of encephalitis lethargica, but states that he was quite well until one day when on parade his eyes became fixed in an upward direction. He is now in a state of Parkinsonism. The pupils are regular, equal, reacting to light but not on accommodation. No convergence. Eye movements jerky. Lid retraction. Blepharoclonus. No blinking on looking to side. His attacks occur daily, usually about two o'clock in the afternoon, just after he has returned from exercise. His eyes turn up, but he can close his eyelids: on doing so his eyes come down, only to go up again on opening his lids. These crises last for about eight hours. The patient can terminate his attacks by going to sleep, and on awaking his eyes are normal. The paroxysms cause him no pain and no visual disturbance.

**Case 3.**—Male, aged 21. Onset of encephalitis, 1924. Now presents typical Parkinsonian appearance, and has respiratory disorder. Pupils equal, regular, reacting to light and not on accommodation. Failure to converge. Double ptosis; eye movements jerky; blepharoclonus. The oculogyral crises in this case were of a peculiar type. They came on chiefly at meal-times. His eyes would turn up, and his eyelids would close tightly. This spasm of the orbiculares palpebrarum would persist for about ten minutes. These attacks occur several times in the course of a day.

**Case 4.**—Male, aged 17. Encephalitis lethargica three and a half years ago; since then has developed a pronounced Parkinsonian condition. Pupils regular, equal, reacting to light but not on accommodation. Convergence poor. Bilateral ptosis. Blinking less frequent, blinks on looking to the side. Blepharoclonus. His attacks last about thirty minutes, during which the eyes are deviated vertically, and with great difficulty the eyes can be brought down. There are no visual hallucinations, but he gets frontal headaches while an attack is in progress. His crises occur two or three times a week.

There are numerous theories as to the pathology of these oculogyral crises. Many observers regard them as entirely functional. Marinesco[12] and Radovici speak of them as "crises hystériformes," as they found that attacks could be precipitated in their patients by injections of sterile water or by watching other patients. Certainly there may be a superimposed functional element in these crises, but the other signs of definite organic disease which occur in the Parkinsonian syndrome do not warrant our calling these attacks hysterical.

The paroxysmal nature of the tonic eye fits makes one think that they are of the same origin as the hyperkinesias, such as spasmodic torticollis, athetosis, etc. These extra-pyramidal hyperkinesias are considered to be due to lesions in the neostriatum. Such is the opinion of Bing, Popowa and Schwartz. Stertz considers there is a striatal centre for conjugate eye movements. According to Bing and Schwartz the hyperkinesias are "release phenomena" due to failure of the inhibitory impulses passing from the neostriatum to the pallidum.

It is also possible that the oculogyral crises are transient irritative phenomena. Although there is no known cortical centre subserving upward ocular deviation, nevertheless a cortical nidus is well known, which, on stimulation, will evoke deviation of the eyes in a lateral direction. One can conceive the lateral oculogyral crises as resulting from stimulation of such a centre. Risien Russell,[13] acting upon the suggestion of Hughlings Jackson, stimulated the cortical oculomotor centre, having previously severed the external rectus muscle of one eye and the internal rectus of the other. The result was a tonic upward movement of the globes. The hypothesis was made of a common oculomotor centre in the cortex, stimulation of which produced—in animals at least—the strongest motor effect, namely deviation in a lateral direction. One can therefore picture these encephalitic oculogyral crises as the result of impulses arising in a previously diseased cortical centre for the representation of eye movements.

I wish to express my thanks to the Honorary Physicians of the Bristol General Hospital and to Dr. Phillips of Southmead Hospital for giving me permission to examine their patients and to publish my observations.

REFERENCES.

1 Duncan, *Brain*, 1924, vol. xlvii., p. 76.

2 Naëf, *Munch. Med. Woch.*, vol. lxvi., 1919.

3 *Report of Local Government Board on Encephalitis Lethargica*, No. 121, London, 1918.

4 Hall, *Lancet*, April 14th, 1923.

5 Kinnier Wilson, Croonian Lecture, *Lancet*, 1925, pp. 1, 53, 169.

6 Chambers, *Brit. M. J.*, 1925, ii. 507.

7 Bing and Schwartz, *L'Encéphale*, No. 3, 1925.

8 A. Wimmer, *Act. Psychet Neur.*, vol. i., Fasc. 2, 1926.

9 Young, *Jour. Neur. and Psycho.*, vol. viii., No. 29, July, 1927.

10 Hohman, *Jour. A. M. A.*, 1925, vol. 84, p. 1,489.

11 Pascheff, *Archiv. d'Ophtalmologie*, No. 12, 1925.

12 Marinesco, cited after Wimmer.

13 Risien Russell, *Journal of Physiol.*, 1894, vol. xvii., p. 1.

